# Prediction of venous thromboembolism incidence in the general adult population using two published genetic risk scores

**DOI:** 10.1371/journal.pone.0280657

**Published:** 2023-01-30

**Authors:** Aaron R. Folsom, Weihong Tang, Ching-Ping Hong, Wayne D. Rosamond, John A. Lane, Mary Cushman, Nathan Pankratz

**Affiliations:** 1 Division of Epidemiology and Community Health, School of Public Health, University of Minnesota, Minneapolis, Minnesota, United States of America; 2 Department of Epidemiology, School of Public Health, University of North Carolina, Chapel Hill, North Carolina, United States of America; 3 Department of Laboratory Medicine and Pathology, University of Minnesota, Minneapolis, Minnesota, United States of America; 4 Department of Medicine and Department of Pathology and Laboratory Medicine, University of Vermont, Burlington, VT, United States of America; Tehran University of Medical Sciences, ISLAMIC REPUBLIC OF IRAN

## Abstract

**Introduction:**

Most strategies for prevention of venous thromboembolism focus on preventing recurrent events. Yet, primary prevention might be possible through approaches targeting the whole population or high-risk patients. To inform possible prevention strategies, population-based information on the ability of genetic risk scores to identify risk of incident venous thromboembolism is needed.

**Materials and methods:**

We used proportional hazards regression to relate two published genetic risk scores (273-variants versus 5-variants) with venous thromboembolism incidence in the Atherosclerosis Risk in Communities Study (ARIC) cohort (n = 11,292), aged 45–64 at baseline, drawn from 4 US communities.

**Results:**

Over a median of 28 years, ARIC identified 788 incident venous thromboembolism events. Incidence rates rose more than two-fold across quartiles of the 273-variant genetic risk score: 1.7, 2.7, 3.4 and 4.0 per 1,000 person-years. For White participants, age, sex, and ancestry-adjusted hazard ratios (95% confidence intervals) across quartiles were strong [1 (reference), 1.30 (0.99,1.70), 1.85 (1.43,2.40), and 2.58 (2.04,3.28)] but weaker for Black participants [1, 1.05 (0.63,1.75), 1.37 (0.84,2.22), and 1.32 (0.80,2.20)]. The 5-variant genetic risk score showed a less steep gradient, with hazard ratios in Whites of 1, 1.17 (0.89,1.54), 1.48 (1.14,1.92), and 2.18 (1.71,2.79). Models including the 273-variant genetic risk score plus lifestyle and clinical factors had a c-statistic of 0.67.

**Conclusions:**

In the general population, middle-aged adults in the highest quartile of either genetic risk score studied have approximately two-fold higher risk of an incident venous thromboembolism compared with the lowest quartile. The genetic risk scores show a weaker association with venous thromboembolism for Black people.

## Introduction

Venous thromboembolism (VTE), consisting of venous thrombosis and pulmonary embolism, is an important cause of morbidity and mortality, with a lifetime risk of approximately one in 12 US adults after age 45 years [1]. One-half to two-thirds of VTEs have provoking factors (i.e., cancer, surgery, trauma, and immobility), whereas the remaining events are considered unprovoked. Advocacy organizations [2] and the US Surgeon General [3] have issued calls to action to prevent VTE, and there are established clinical guidelines and methods for acute treatment, secondary prevention, and prevention of provoked VTE in high-risk patients [2, 4–8].

Current strategies for preventing VTE fail to address primary prevention of unprovoked VTE in the general population [9]. One approach to primary prevention of unprovoked VTE would be a population-wide strategy using education, environmental changes, and policy to reduce modifiable VTE risk factors. Another would be to screen individuals for VTE risk factors and target safe interventions toward high-risk individuals. Yet, identifying individuals at high risk for VTE is challenging, as there are few published, feasible risk scores to identify those in the general population at substantial risk of incident VTE.

Risk scores for screening the general population might assess lifestyle or clinical risk factors; however, except for age and obesity, many traditional arterial cardiovascular risk factors (e.g., hypertension, diabetes, hyperlipidemia, and smoking) are, at most, weak VTE risk factors [9]. We have shown, for example, that the American College of Cardiology-American Heart Association pooled risk equation for cardiovascular disease does not predict VTE in the Atherosclerosis Risk in Communities (ARIC) study [9]. On the other hand, a poor American Heart Association’s Life’s Simple 7 (LS7) score is associated with greater incident VTE [10–12], largely by the inclusion of obesity in LS7.

Genetic risk scores (GRSs) can also predict VTE in the general population. In a Dutch case-control study, de Haan et al. derived a 5-variant GRS of hemostatic factor variants (composed of *F5* Leiden rs6025, *F2* rs1799963, *ABO* rs8176719 [O versus non-O groups], *FGG* rs2066865, and *F11* rs2036914) and showed that the risk of VTE was significantly higher for each additional variant in the GRS [13]. The 5-variant GRS (c-statistic = 0.68) predicted incident VTE better that *F5* Leiden alone did. This 0.68 c-statistic was lower than the c-statistic of 0.77 for a nongenetic risk score composed of clinical and provoking factors and 0.82 for the combined genetic plus nongenetic score. We replicated the positive association of this 5-variant GRS with VTE, beyond *F5* Leiden alone, in White but not Black individuals in ARIC [14], and independent of LS7 score [15].

Klarin et al. recently reported that a GRS comprising 297 variants strongly predicted VTE prospectively in several large cohorts [16]. Those in the upper 5% of the population on the 297-variant risk score had an incident VTE risk equivalent to carriers of *F5* Leiden (R506Q) or *F2* rs1799963. Klarin et al. explicitly excluded *F5* Leiden or *F2* rs1799963 and any variant in strong linkage disequilibrium (r^2^>0.20) from their 297-variant GRS. However, the 297-variant GRS included 36 other variants at the *F5* locus and 6 other variants at the *F2* locus that are collectively tagging these two highly penetrant variants quite well (D’ = 1.0, despite having r^2^<0.20), and so the GRS may still be reflecting risk from *F5* Leiden or the *F2* rs1799963. Thus, the 297-variant GRS should be tested in additional populations against the 5-variant GRS, head-to-head, and jointly with the 5-variant GRS.

Our aims were (a) to assess the degree to which the most comprehensive published VTE GRS, that of Klarin et al., predicts VTE incidence in the prospective ARIC Study, (b) to compare it with the simplest published VTE GRS, the 5-variant GRS of de Haan et al., in Black and White participants, and (c) to test the combined prediction of VTE using the GRSs plus socioeconomic, clinical and lifestyle risk factors.

## Materials and methods

### Study sample and design

Previous publications described the overall ARIC study design, methods, and VTE incidence rates in detail [17, 18]. Briefly, in 1987–1989 ARIC recruited 15,792 predominantly Black or White men and women aged 45 to 64 years from four US communities—Forsyth County, NC; Jackson, MS (Blacks only); suburban Minneapolis, MN; Washington County, MD. After participants gave informed consent, ARIC performed a baseline (Visit 1) examination, which included genetic testing on stored DNA. ARIC maintained longitudinal contact with the participants via annual or semi-annual telephone calls and 6 reexamination visits conducted from 1990–2019. Prior to study onset, the institutional review committees at each ARIC field center (University of Minnesota, University of Mississippi, Johns Hopkins University, Wake Forest University) and coordinating center (University of North Carolina) approved the protocols for ARIC and this study of VTE. All participants provided written informed consent.

### Measurement of genomic variants at ARIC baseline

ARIC isolated genomic DNA from buffy coat specimens. The ARIC DNA laboratory at the University of Texas at Houston genotyped the variants for the 5-variant GRS reported by de Haan et al [13]: *F5* rs6025 and *FGG* rs2066865 using the iPLEX multiplex assay that utilizes the MassARRAY system (Sequenom, Inc., San Diego, CA), *ABO* rs8176719 using the functionally tested TaqMan Assay-by-Design system (Applied Biosystems, Foster City, CA), *F2* rs1799963 using the pre-validated TaqMan Assay-on-Demand system (Applied Biosystems, Foster City, CA), and *F11* rs2036914 using the ITMAT-Broad-CARe custom array (Illumina, San Diego, CA) [19]. To control for population stratification in race-specific models, we estimated and adjusted for ten principal components of ancestry using Eigenstrat [20] with genotypes from the Affymetrix Genome-wide Human SNP array 6.0.

GRSs combine signals from multiple loci by summing the product of the effect size computed by an external source and the number of effect alleles (0, 1, or 2 for genotyped markers; dosage values for imputed markers) at each locus for each participant. In this way, alleles with a larger reported effect on VTE risk will have a larger contribution to the final score. We computed the weighted 5-variant score using the effect sizes reported by de Haan et al. as the weights [13, 14]. Because the 5-variant score based on ARIC original genotyping had substantial missing data (n = 798 missing) [14], for this report, we used TOPMed reference panel version r1, available from the TOPMed imputation server (https://imputation.biodatacatalyst.nhlbi.nih.gov/#!pages/about), to impute values missing from the Affymetrix Genome-wide Human SNP array 6.0 genotypes, after standard quality control measures described previously [21] For those with both an original and an imputed 5-variant GRS, the Pearson correlation of the original classification with the imputed classification was high, ranging from r = 0.95 to 1.00 for the 5 individual variants and being r = 0.88 for the overall 5-variant GRS.

The TOPMed imputation reference panel r1 contained 273 of the 297 variants in the GRS of Klarin et al., [16] and we used the published weights and the algorithm outlined above to create a weighted 273-variant GRS for this report (see **S1 Table** for which variants were included or not included) [16]. We attempted to identify proxies for the 24 missing variants based on linkage disequilibrium (r^**2**^>0.80) using publicly available sequencing data. We were able to find a set of proxies for 12 of them; however, all but three of them failed TOPMed quality control and were therefore not included on the TOPMed r1 panel, which meant that they could not be imputed in this study. We therefore focused analyses on the directly-measured, 273-variants of the GRS. We created two additional GRSs for sensitivity analyses: 1) a 160-variant GRS that excluded any of the available 273 variants from Klarin et al. that were physically near any of the five variants from de Haan et al. (variants removed from the sensitivity analysis are flagged in the “de Haan Region” column of S1 and S2 Tables) a GRS comprising residuals of the Klarin 273-variant score after regressing out all five de Haan variants via multiple linear regression.

### Measurement of clinical and lifestyle risk factors

Using methods previously reported [11, 22, 23], this analysis included factors that proved to be VTE risk factors in ARIC, assessed at the ARIC baseline examination: age (years), race (White, Black), sex, hormone replacement therapy (current, former, never), education level (<high school, high school grad, >high school grad), household income (<$12,000, $12,000 to $24,999, $25,000 to $49,999, $50,000+, missing), body height and weight, and estimated glomerular filtration rate. We also included other major non-lipid cardiovascular risk factors measured at baseline, even though they are, at best, weakly associated with VTE in ARIC: diabetes (yes defined as fasting serum glucose ≥126 mg/dL from a single blood draw, medication, or physician diagnosis; no), smoking status (current, former, never), sports physical activity level (continuous variable—questionnaire index of frequency and duration of habitual sports [22]), systolic blood pressure (continuous variable—average of the last two of three seated measurements, after five minutes rest), and antihypertensive medication use (yes, no).

### Identification of incident VTE

After the Visit 1 examination, ARIC staff telephoned participants, initially annually then semi-annually from 2012, and asked about all hospitalizations in the previous year. Staff then obtained and recorded in-hospital ICD-9-CM codes for all discharge diagnoses and copied selected hospital record material for validation of VTE through 2019. To validate VTE events, two physicians reviewed the records using standardized criteria [18] requiring positive imaging tests for diagnosis of clinically recognized DVT and PE. The physicians sub-classified VTEs as provoked (associated with cancer, major trauma, surgery, or marked immobility) or unprovoked (none of these causes). For this report, we restricted DVTs to those in the lower extremity or vena cava, because upper extremity DVTs were relatively few and almost always the result of indwelling venous catheters.

### Statistical methods

From the ARIC baseline cohort (*n* = 15,792), we successively excluded 48 who were not Black or White, 276 who reported a history of VTE at baseline, 71 who were taking anticoagulants at baseline, 3,456 without the Affymetrix 6.0 array data needed to create the Klarin et al. GRS (i.e., declined DNA use, no usable DNA, etc.), 467 with missing covariables for Model 2, 92 with missing covariables for Model 3, and 90 with missing principal components of ancestry, which left 11,292 participants with complete data for analysis. As shown in **S2 Table**, the baseline characteristics of those included in the analysis were nearly identical to the whole cohort’s characteristics; the largest difference was that fewer Blacks (22%) were in the analytic sample than in the original ARIC cohort (27%). The proportion who developed VTE was also nearly identical for the analytic sample (7.0%) compared with the original ARIC cohort (7.2%).

Using SAS, we first compared the 273-variant GRS of Klarin et al. with the 5-variant GRS of de Haan et al. by computing the shared variance between the two scores and by computing the mean 273-variant score by the number of alleles present for 5-variant score. We computed crude incidence rates of VTE (number of incident events divided by person-years of follow-up) in relation to quartiles of the GRSs in the entire ARIC sample. Follow-up for person-year calculation began at ARIC baseline and continued until the date of first VTE, loss to follow-up, death, or December 31, 2019. We also created Kaplan-Meier plots of the cumulative incidence of VTE by GRS quartiles.

We performed proportional hazards regression to estimate the hazard ratios (HR) of VTE in relation to each GRS, including the two supplemental GRSs. In the past, we verified that the proportional hazards assumption held for age and sex for many variables by testing interactions with follow-up time, by demographic-specific plots of the survival function over time, and by correlating Schoenfeld residuals and the ranking of individual failure times. However, we have consistently found that the proportional hazards assumption for race was violated, in that Blacks had an increasingly greater risk of VTE, compared with Whites, as follow-up lengthened [23]. To account for this and the fact that associations with GRSs were stronger for Whites than Blacks, we present many of the main associations stratified by race. We adjusted the race-specific HRs for baseline age and sex, and the principal components of ancestry (Model 1). We also created restricted cubic spline plots using Model 1. In Model 2, we added other baseline socioeconomic, clinical and lifestyle factors, and calculated Harrell’s c-statistic as a measure of model prediction (i.e., discrimination) for Models 1 and 2.

In addition, we determined the following in the whole ARIC cohort: (1) whether the 273-variant GRS and 5-SNP variant GRS were independently associated with VTE when adjusted for each other and (2) the degree to which VTE incidence rates were associated with a combined score using the quartiles of GRSs jointly.

## Results

### Descriptive baseline characteristics of the ARIC sample and of the GRSs

The 11,292 ARIC participants in this analysis were 45–64 years old at baseline in 1987–89; 54% were women, 78% were Whites, and 22% were Blacks (**S2 Table**). The participants had median scores of 21.4 (range = 17.4 to 28.5) for the 273-variant GRS and 1.0 (range = 0 to 4.2) for the imputed, weighted 5-variant GRS. The mean 273-variant score rose for each higher allele group of the 5-variant score (**S1 Fig**), and in a multiple regression model the 5-variant GRS explained 32.6% of the variance of the 273-variant GRS.

As derived by Klarin et al., [16] the 273-variant GRS did not include *F5* Leiden rs6025 or *F2* rs1799963, two well-known VTE risk variants included in the 5-variant GRS. Yet, even so, ARIC participants in the highest 5% of the 273-variant GRS still had a much higher frequency of *F5* Leiden rs6025 (39%) than those in the lowest 95% of the distribution (5%), and a full 75% of those in the highest 1% of scores carried *F5* Leiden. Expressed in a different way, more than half of all *F5* Leiden carriers were in the top 10% of the 273-variant score distribution. Overall, the number of *F5* Leiden alleles explained 9.8% of the total variance in the 273-variant GRS (see **S2 Fig** for the average 273-GRS score for those with 0, 1 or 2 copies of *F5* Leiden).

The frequencies of *F2* rs1799963 were similar in the highest 5% and lowest 95% (22% and 23%, respectively). Given how rare the *F2* rs1799963 is, the number of alleles for this variant explained only 0.045% of the total variance in the 273-variant GRS.

### Association of 273-variant GRS with incident VTE overall and race-specific comparison with the 5-variant GRS

During a median of 28 years of follow-up (maximum 33 years), ARIC ascertained 788 participants with an incident VTE (i.e., deep vein thrombosis of the lower extremity or a pulmonary embolism). Of these, 467 were provoked and 321 were unprovoked. Participants with higher scores on the 273-variant GRS had higher risk of total VTE, with VTE incidence rates in the whole cohort being 1.7, 2.7, 3.4, and 4.0 per 1,000 person-years across the quartiles of the GRS (**Table 1** and **S3 Fig**). The overall VTE rate was higher in Black participants than White participants, but the gradient of risk across quartiles was steeper in White participants. The associations appeared slightly stronger for unprovoked than provoked VTE.

**Table 1:** Incidence rates and race-specific hazard ratios (HR) for venous thromboembolism (VTE) in relation to quartiles of the 273-variant genetic risk score (GRS), ARIC*, 1987–2019.

GRS quartile	1	2	3	4
VTE events (n)**†**	121	164	230	273
Person years	69,581	68,982	67,342	67,511
**VTE Incidence** per 10^3^	1.7	2.4	3.4	4.0
**VTE Incidence for White participants**	1.6	2.0	2.9	3.9
**HR (95% CI) for White participants**				
Total VTE (Model 1) ^**‡**^	1 (Reference)	1.30 (0.99,1.70)	1.85 (1.43,2.40)	2.58 (2.04,3.28)
Unprovoked VTE^**‡**^	1 (Reference)	1.18 (0.73,1.92)	2.52 (1.65,3.86)	3.81 (2.57,5.65)
Provoked VTE^**‡**^	1 (Reference)	1.35 (0.98,1.88)	1.53 (1.10,2.12)	2.00 (1.47,2.70)
Total VTE (Model 2)**¶**	1 (Reference)	1.26 (0.96,1.65)	1.82 (1.40,2.35)	2.52 (1.99,3.20)
Unprovoked VTE**¶**	1 (Reference)	1.15 (0.71,1.88)	2.48 (1.62,3.79)	3.74 (2.52,5.54)
Provoked VTE**¶**	1 (Reference)	1.30 (0.94,1.81)	1.50 (1.08,2.08)	1.93 (1.43,2.62)
**VTE Incidence for Black participants**	3.5	3.6	4.6	4.4
**HR (95% CI) for Black participants**				
Total VTE (Model 1) ^**‡**^	1 (reference)	1.05 (0.63,1.75)	1.37 (0.84,2.22)	1.32 (0.80,2.20)
Unprovoked VTE^**‡**^	1 (Reference)	0.80 (0.34, 1.86)	1.55 (0.73,3.31)	1.43 (0.65, 3.17)
Provoked VTE^**‡**^	1 (Reference)	1.21 (0.63,2.31)	1.24 (0.66,2.32)	1.25 (0.65,2.41)
Total VTE (Model 2)**¶**	1 (Reference)	1.08 (0.65, 1.81)	1.37 (0.84,2.23)	1.35 (0.81,2.25)
Unprovoked VTE**¶**	1 (Reference)	0.83 (0.36, 1.94)	1.55 (0.72,3.32)	1.50 (0.68, 3.34)
Provoked VTE**¶**	1 (Reference)	1.24 (0.65,2.39)	1.24 (0.66,2.34)	1.25 (0.65,2.43)

*At baseline in 1987–89

**†**Total VTE n = 788; unprovoked VTE n = 321; provoked VTE n = 467.

^**‡**^Model 1: Adjusted for age, sex, and principal components of ancestry.

**¶**Model 2: Adjusted for age, sex, principal components of ancestry, hormone replacement therapy (current, former, never for women, with men as referent category), education level (<high school, high school grad, >high school grad), household income (<$12,000, $12,000 to $24,999, $25,000 to $49,999, $50,000+, missing), height (continuous), weight (continuous), estimated glomerular filtration rate (continuous), diabetes (yes defined as >126 mg/dL, medication or physician diagnosis; no), smoking status (current, former, never), sports physical activity level (continuous), systolic blood pressure (continuous), antihypertensive medication use (yes, no)

Abbreviation: ARIC = Atherosclerosis Risk in Communities Study

**Fig 1:**
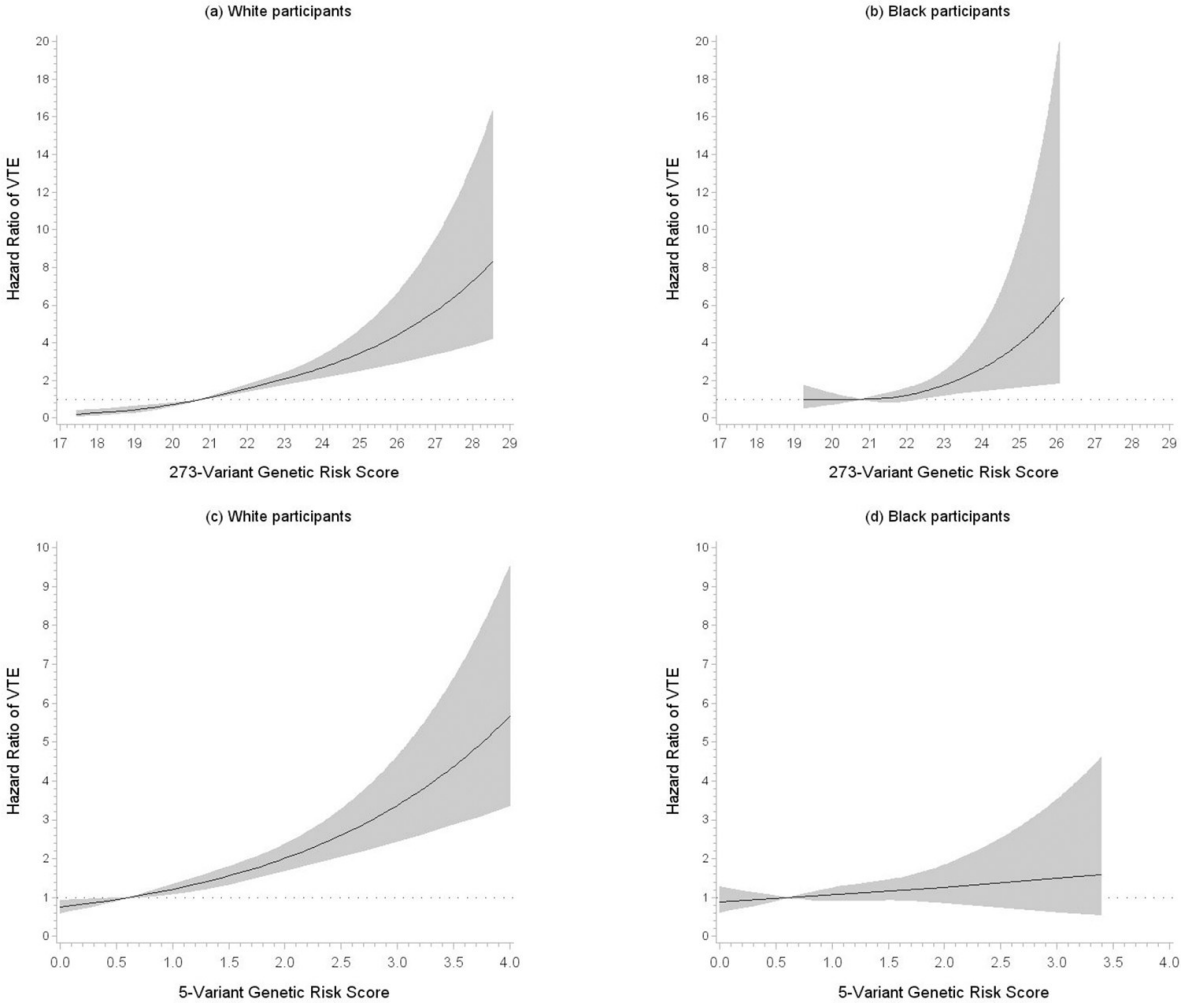
Hazard ratios of venous thromboembolism in relation to the 273-variant genetic risk score (GRS) in (a) White participants and (b) Black participants or the 5-Variant GRS in (c) White participants and (d) Black participants, ARIC, 1987–2019. Created using restricted cubic spline model with 3 knots and adjusted for age, sex, and principal components of ancestry. Reference values for the hazard ratios were 20.8 for the 273-variant score and 0.61 for the 5-variant score.

**Fig 1** depicts the race-specific continuous relation of the 273-variant GRS with VTE, which was stronger for White than Black participants. In White participants (n = 553 VTEs), HRs adjusted for age, sex, and principal components of ancestry (Model 1, **Table 1**) were: 1 (reference), 1.30 (0.99,1.70), 1.85 (1.43,2.40), and 2.58 (2.04,3.28). The graded, positive association was stronger for unprovoked VTE than for provoked VTE. In contrast, in Black participants, the Model 1 HRs of total VTE (n = 235 VTEs) were minimally increased with no trend across quartiles and not statistically significant: 1 (reference), 1.05 (0.63,1.75), 1.37 (0.84,2.22), and 1.32 (0.80,2.20). The c-statistic for Model 1 was 0.64 for White participants and 0.61 for Black participants. Additional inclusion of baseline socioeconomic, lifestyle and clinical risk factors (Model 2, **Table 1**) did not appreciably alter the HRs of total VTE in either White or Black participants but slightly improve the prediction (i.e., discrimination) of VTE; the c-statistic for Model 2 rose to 0.67 for both Black and White participants.

The 5-variant GRS also showed a significant positive association with VTE incidence (**Fig 1**), but HRs in Whites were somewhat weaker compared with the 273-variant GRS. As shown in **Table 2**, in White participants, the Model 1 adjusted HRs (95% CIs) of total VTE across quartiles of the 5-variant GRS were: 1 (reference), 1.17 (0.89,1.54), 1.48 (1.14,1.92), and 2.18 (1.71,2.79). In Black participants, the HRs were minimally increased without a trend and not significant: 1 (reference), 1.39 (0.96,2.01), 1.25 (0.87,1.81), and 1.32 (0.90,1.93). The c-statistic for Model 1 was 0.63 for White participants and 0.61 for Black participants. The HRs for Model 2 were almost the same as for Model 1, but the c-statistic for Model 2 rose to 0.66 for White participants and 0.67 for Black participants, which suggests adding in the baseline socioeconomic, lifestyle, and clinical risk factors modestly improved prediction of VTE beyond the GRS itself. The associations appeared slightly stronger for unprovoked than provoked VTE.

**Table 2:** Incidence rates and race-specific hazard ratios (HR) for venous thromboembolism (VTE) in relation to quartiles of the 5-variant genetic risk score (GRS), ARIC*, 1987–2019.

GRS quartile	1	2	3	4
VTE events (n) **†**	146	177	202	263
Person years	69,016	68,338	67,947	68,114
**VTE Incidence** per 10^3^	2.1	2.6	3.0	3.9
**VTE Incidence for White Participants**	1.8	2.1	2.6	3.7
**HR (95% CI) for White Participants**				
Total VTE (Model 1) ^**‡**^	1 (Reference)	1.17 (0.89,1.54)	1.48 (1.14,1.92)	2.18 (1.71,2.79)
Unprovoked VTE^**‡**^	1 (Reference)	1.28 (0.81,2.01)	1.86 (1.22,2.85)	2.65 (1.78,3.96)
Provoked VTE^**‡**^	1 (Reference)	1.12 (0.80,1.58)	1.28 (0.91,1,79)	1.94 (1.42,2.64)
Total VTE (Model 2)**¶**	1 (Reference)	1.19 (0.91,1.56)	1.49 (1.14,1.94)	2.18 (1.71,2.78)
Unprovoked VTE**¶**	1 (Reference)	1.30 (0.83,2.05)	1.90 (1.24,2.90)	2.68 (1.80,4.00)
Provoked VTE**¶**	1 (Reference)	1.13 (0.81,1.59)	1.28 (0.91,1.79)	1.91 (1.40,2.60)
**VTE Incidence for Black Participants**	3.4	4.6	4.2	4.5
**HR (95% CI) for Black Participants**				
Total VTE (Model 1) ^**‡**^	1 (reference)	1.39 (0.96,2.01)	1.25 (0.87,1.81)	1.32 (0.90,1.93)
Unprovoked VTE ^**‡**^	1 (Reference)	1.53 (0.84,2.76)	1.47 (0.82,2.63)	1.39 (0.75,2.58)
Provoked VTE ^**‡**^	1 (Reference)	1.30 (0.81,2.10)	1.13 (0.71,1.81)	1.27 (0.78,2.06)
Total VTE (Model 2)†	1 (Reference)	1.35 (0.93,1.97)	1.23 (0.85,1.77)	1.36 (0.93, 1.99)
Unprovoked VTE**¶**	1 (Reference)	1.49 (0.82,2.71)	1.37 (0.76,2.47)	1.46 (0.78,2.71)
Provoked VTE**¶**	1 (Reference)	1.29 (0.80,2.08)	1.14 (0.71,1.84)	1.31 (0.81,2.14)

*At baseline in 1987–89

**†**Total VTE n = 788; unprovoked VTE n = 321; provoked VTE n = 467.

^**‡**^Model 1: Adjusted for age, sex, and principal components of ancestry.

**¶**Model 2: Adjusted for age, sex, principal components of ancestry, hormone replacement therapy (current, former, never for women, with men as referent category), education level (<high school, high school grad, >high school grad), household income (<$12,000, $12,000 to $24,999, $25,000 to $49,999, $50,000+, missing), height (continuous), weight (continuous), estimated glomerular filtration rate (continuous), diabetes (yes defined as >126 mg/dL, medication or physician diagnosis; no), smoking status (current, former, never), sports physical activity level (continuous), systolic blood pressure (continuous), antihypertensive medication use (yes, no)

Abbreviation: ARIC = Atherosclerosis Risk in Communities Study

### Race-specific association of the two supplemental GRSs with total VTE

Even though Klarin et al. excluded *F5* Leiden rs6025, *F2* rs1799963, and all variants that were in even moderate linkage disequlibrium with them (r^2^>0.20) from their 297-variant GRS, their GRS still included many variants linked to *F5* and *F2* (as described above). We therefore recalculated VTE HRs using our supplemental GRSs to determine whether the Klarin et al. GRS mainly predicted VTE because it contained other predictive variants linked to those in the 5-variant score. As shown in **Table 3**, in White participants, the HRs for VTE were substantially weaker for the 160-variant GRS that excluded all measured variants physically near any variant in the 5-variant score, and the HRs were moderately weaker for the GRS using residuals of the Klarin 273-variant score after regressing out just the five de Haan variants. In Black participants, for whom the sample size was smaller and the association of VTE with the main 273-variant GRS was weak (Table 1), the HRs using the supplemental GRSs were essentially null (**Table 3**).

**Table 3:** Race-specific hazard ratios (HR) for venous thromboembolism (VTE) in relation to quartiles of the 273-variant genetic risk score (GRS) versus two supplemental GRSs, ARIC*, 1987–2019.

GRS quartile	1	2	3	4	c-statistic
**HR (95% CI) of total VTE** † **for White participants**					
273- variant GRS (per Table 1)	1 (Reference)	1.30 (0.99,1.70)	1.85 (1.42,2.40)	2.58 (2.04,3.28)	0.64
160-variant GRS^**‡**^	1 (Reference)	1.02 (0.81,1.30)	1.33 (1.05,1.67)	1.66 (1.31,2.10)	0.61
273-variant residuals GRS**¶**	1 (Reference)	1.34 (1.04,1.72)	1.43 (1.11,1.84)	2.04 (1.61,2.59)	0.62
**HR (95% CI) of total VTE** † **for Black participants**					
273- variant GRS (per Table 1)	1 (reference)	1.05 (0.63,1.75)	1.37 (0.84,2.22)	1.32 (0.80,2.20)	0.61
160-variant GRS^**‡**^	1 (Reference)	0.90 (0.42,1.92)	1.36 (0.68,2.73)	1.28 (0.64, 2.53)	0.61
273-variant residuals GRS**¶**	1 (Reference)	0.69 (0.44,1.08)	0.84 (0.56,1.27)	1.06 (0.71,1.58)	0.61

*At baseline in 1987–89

**†**Model 1: Adjusted for age, sex, and principal components of ancestry.

^‡^After excluding from the 273-variant GRS all variants physically near variants in the 5-variant GRS

¶273-variant GRS, after regressing out all five variants in the 5-variant GRS

### Independent and joint associations of GRSs with VTE in the ARIC cohort

Even though there is some overlap between the 273-variant and 5-variant GRSs, we were curious whether together they might predict VTE better than either alone. When the two GRSs were adjusted for each other in the same model containing both White and Black participants, the age, race, and sex-adjusted HRs for each were attenuated but still the associations were stronger for the 273-SNP score. The jointly adjusted HRs across quartiles were: 1 (reference), 1.24 (95% CI 0.97,1.57), 1.64 (1.30,2.08) and 1.94 (1.53,2.47) for the 273-variant GRS versus 1 (reference), 1.12 (95% CI 0.90,1.40), 1.15 (0.92,1.43), and 1.39 (1.11,1.75) for the 5-variant GRS. HRs for White and Black participants separately are shown in S3 Table.

We also computed the VTE incidence rates of ARIC participants simultaneously in the highest quartiles of both GRSs versus those in the lowest quartiles of both. For those in the highest joint GRS group, the age, race, and sex-adjusted incidence rate [4.6 (95% CI 4.0, 5.4) per 1,000 person-years] was nearly 3-times higher than the rate for those in the lowest joint GRS group [1.6 (95% CI 1.2, 2.1).].

## Discussion

In this large, community-based, prospective study of middle-aged adults followed for more than three decades, we corroborated that the polygenic risk score created by Klarin et al. [16] discriminated moderately well between those at higher and lower risk of incident VTE, particularly among White participants. The incidence rate was approximately double for the highest versus lowest quartile of the GRS and was stronger for unprovoked than provoked VTE, as anticipated for a GRS. The 273-variant GRS showed a stronger association with VTE than did the 5-variant GRS created by de Haan et al., [13] although the simpler 5-variant GRS, by itself, was strongly associated with VTE.

We were able to include 273 of the 297 variants that Klarin et al. derived for their GRS for VTE. The 24 remaining variants were not included in the TOPMed reference panel used to impute this GRS (**S1 Table**) due to quality control issues. Although proxies exist for these missing variants, nearly all of those proxies were excluded from the TOPMed reference panel for quality control concerns as well.

Klarin et al. intentionally excluded two powerful genetic variants for VTE that are included in the 5-variant GRS, namely *F5* Leiden rs6025 and *F2* rs1799963, which allowed Klarin et al. to demonstrate that having a 297-variant GRS score above the 95^th^ percentile predicted VTE as well as the presence of the *F5* and *F2* variants did [16] Yet, we verified in ARIC that the 273-variant GRS contains several other *F5* variants capturing *F5* Leiden, such that 39% of those above the 95^th^ percentile of the 273-variant GRS actually carried *F5* Leiden. The fact that the 5-variant GRS accounted for 32.6% of the variance in the 273-variant GRS also suggested overlap between the two GRSs. The HRs of VTE across GRS quartiles were weaker for our two supplemental GRSs, compared to the 273-variant GRS in White participants, further suggesting that the de Haan variants contribute to VTE prediction by the Klarin et al. GRS, as do additional variants physically near the five de Haan variants. Since the supplemental GRS that regressed out the five variants had a stronger association with VTE than the one that removed all variants from these loci, this suggests that there are independent signals at these five loci that are contributing to risk, such as the independent rs4524 missense variant in F5 and three other independent signals at ABO [24]. This stresses the importance of performing conditional analyses in discovery analyses that are based on regressing out signals instead of relying on linkage disequilibrium.

When we alternatively adjusted the 273-variant and 5-variant GRSs for one another, the two scores had partly shared but partly independent associations with VTE. Hence, if one wanted to use genetic variants to identify people at risk of VTE incidence, the two scores together predict VTE somewhat better than either one did by itself. Either GRS predicts VTE better than *F5* Leiden alone [13, 16].

Black participants had higher VTE incidence rates than White participants. The association of each GRS with VTE was strong and significant for White participants but not for Black individuals, as we previously reported for the 5-variant GRS with shorter ARIC follow-up time and fewer VTEs [14]. Some possible reasons for this are that, firstly, there were fewer Black participants than White, leading to less precision. Secondly, the populations from which the VTE GRSs were derived were overwhelmingly White and therefore GRSs were not optimized for Black persons. Thirdly, both GRSs are moderately influenced by *F5* Leiden, which is the strongest common genetic risk factor for VTE and is five times less common in Black than White people. Finally, because ARIC Black participants had a higher baseline VTE rate than White participants, it is plausible that genetic status conveyed less additional hazard of VTE in Black than White participants. Future research should include large studies to identify causal variants for VTE in populations of color.

The associations of both GRSs with VTE remained strong after adjustment for multiple clinical and lifestyle risk factors for VTE measured at ARIC’s baseline examination. This was expected, as generally there is little obvious correlation of lifestyle and clinical factors with VTE genetic variants.

Limitations of our study warrant consideration. Firstly, the ARIC study was part of the INVENT consortium [24], which was one of several data sets that Klarin et al. used to externally validate their GRS. However, our study offers considerably more detail on the generalizability of the 273-variant GRS to Black persons, directly compares to a much simpler 5-variant GRS, and this analysis incorporated longer ARIC follow up with more VTE cases. Secondly, researchers have pointed out limitations to GRS modeling to date and the need for better models [25, 26]. Our aim was not to derive a new GRS but rather to compare two existing GRSs, that with the least variants (n = 5) and that with the most variants (n = nearly 300), for Black and White participants. Thirdly, ARIC was unable to obtain information on outpatient treated VTEs, which early in the three decades of follow-up were rare but certainly were more common in recent years. Loss of these events is unlikely to lead to significant bias in prediction, as genetic risk score seems unlikely to be appreciably related to whether a DVT was diagnosed in the hospital vs. outpatient setting. Finally, ARIC participants were at least 45 years of age at recruitment, and so our results may not generalize to individuals younger than 45. Finally, to study incident VTE, we excluded 347 ARIC participants at baseline who had a history of self-reported, unvalidated VTE or were using anticoagulants. Those excluded participants may have had a greater genetic predisposition to VTE and thus the association of the GRS with incident VTE may underestimate the actual association.

Many researchers have derived GRSs to identify patients at potential risk of various diseases. Yet, the clinical utility of GRSs in the general population is largely unevaluated or uncertain, even though the future prospect of measuring genomic risk of multiple diseases early in life to guide prevention across the life-course is appealing. Currently, VTE prevention and treatment guidelines do not advocate incorporation of GRS information. We sought to further evaluate the validity and generalizability of two prominent VTE GRSs in a diverse general population. Regarding VTE prediction, our Model 1 c-statistics of 0.63 in White participants and 0.61 in Black participants were smaller than the 0.68 value reported for the 5-variant GRS derived by the Dutch case-control study of de Haan et al. [13] de Haan et al. also reported their c-statistic to be 0.82 when clinical and provoking factors measured just before VTE were added to their model. In our prospective cohort study, the addition of non-genetic factors measured increased the c-statistics modestly to 0.66–0.67. Possible reasons for the lower c-statistics in ARIC are: firstly, the de Haan study derived the GRS and had no validation sample, and risk prediction is nearly always stronger in a derivation sample than a validation sample; secondly, de Haan chose genetic variants from a presumably homogeneous Dutch study population, whereas the ARIC validated the GRS in a diverse US population; thirdly, our cohort had a long follow-up for VTE, which likely would attenuate prediction by clinical risk factors compared with a case-control study, where clinical risk factors in cases were measured close to VTE occurrence.

At least four additional publications have also created GRSs for VTE, containing between 7 and 37 variants [24, 27–29], with VTE prediction similar to the 5-variant and 273-variant GRSs in our study. Most notably, using the enormous UK Biobank cohort, Kolin et. al. [29] created a GRS of 36-variants to predict VTE, yielding a crude c-statistic of 0.62 and a combined GRS/clinical score c-statistic of 0.69. The 273-variant that we studied had most of the 36 variants of Kolin et. al. and in whites yielded similar crude and clinical risk factor adjusted c-statistics (0.64 and 0.67, respectively). The 5-variant GRS yielded only slightly lower c-statistics for VTE prediction in our ARIC cohort. If a GRS, or a GRS plus clinical risk factor score, were to be considered for predicting VTE in the general population, a GRS with just a few dozen variants might be more practical than a GRS with several hundred variants. Yet, it is crucial to verify that any GRS or clinical risk score for VTE predicts well in the intended population.

## Conclusions

In the general population, middle-aged adults in the highest quartile of either genetic risk score studied have approximately a two-fold higher risk of an incident VTE event compared with those in the lowest quartile. The genetic risk scores however show a weaker association with venous thromboembolism for Black people. Whether primary prevention of incident VTE is feasible in a general population by either a high-risk patient or a population strategy needs further exploration.

## Supporting information

S1 Table (XLSX)

S2 Table Baseline characteristics (mean or percent) of the full cohort compared with the cohort included in the analysis, ARIC, 1987–89. (DOCX)

S3 Table Race-specific hazard ratios (HR) for venous thromboembolism (VTE) in relation to quartiles of the 273-variant and the 5-variant genetic risk scores (GRS) examined jointly in model 1, ARIC*, 1987–2019. (DOCX)

S1 Fig (TIFF)

S2 Fig (TIFF)

S3 Fig Cumulative incidence of venous thromboembolism (VTE) by quartiles of (a) the 273-variant genetic risk score (GRS) or (b) the 5-variant GRS, ARIC, 1987–2019. (DOCX)
