# Supplementary material for: Prediction of venous thromboembolism incidence in the general adult population using two published genetic risk scores
Source: PLoS One. 2023 Jan 30;18(1):e0280657. doi: 10.1371/journal.pone.0280657 (PMC9886242; doi:10.1371/journal.pone.0280657)
Supplement: S2 Table — (DOCX) [file pone.0280657.s002.docx]

**Supplemental Table S2**. Baseline characteristics (mean or percent) of the full cohort compared with the cohort included in the analysis, ARIC, 1987-89

Characteristic Full Sample Included Sample

_____________________________________________________________________________

| n |  | 15,511-15,792* | 11,292 |
| --- | --- | --- | --- |
|  |  |  |  |
| Age, years | | 54 | 54 |
| Sex, % | |  |  |
|  | Male | 46 | 46 |
|  | Female | 54 | 54 |
| Race, % | |  |  |
|  | White | 73 | 78 |
|  | Black | 27 | 22 |
| Education <high school graduate, % | | 24 | 21 |
| Household income <$12,000, % | | 16 | 13 |
| Current hormone replacement therapy, % of women | | 19 | 20 |
| Height, cm | | 168 | 168 |
| Weight, pounds | | 173 | 172 |
| Sport index (range 0-5) | | 2.4 | 2.4 |
| Systolic BP, mm Hg | | 121 | 121 |
| Estimated glomerular filtration rate, mL/min/1.73m^2^ | | 102 | 102 |
| Current smoker, % | | 26 | 26 |
| Diabetes, % | | 12 | 11 |
| Antihypertensive medication, % | | 25 | 25 |
| Factor VIII, % | | 131 | 129 |
| von Willebrand factor, % | | 118 | 116 |
| Protein C, mg/L | | 3.2 | 3.2 |
| Activated partial thromboplastin time, sec | | 29 | 29 |
|  | |  |  |

^_________________________________________________________________________________________________________________
*^Sample size varies among variables.

Abbreviation: ARIC = Atherosclerosis Risk in Communities Study
