# Supplementary material for: Prediction of venous thromboembolism incidence in the general adult population using two published genetic risk scores
Source: PLoS One. 2023 Jan 30;18(1):e0280657. doi: 10.1371/journal.pone.0280657 (PMC9886242; doi:10.1371/journal.pone.0280657)
Supplement: S3 Table — (DOCX) [file pone.0280657.s003.docx]

**Supplemental Table S3. Race-specific hazard ratios (HR) for venous thromboembolism (VTE) in relation to quartiles of the 273-variant and the 5-variant genetic risk scores (GRS) examined jointly in model 1, ARIC*, 1987-2019**

| GRS quartile | 1 | 2 | 3 | 4 |
| --- | --- | --- | --- | --- |
| **HR (95% CI) of total VTE ^‡^ for All participants**  273-variant GRS | 1 (Reference) | 1.24 (0.97,1.57 | 1.64 (1.30,2.08) | 1.94 (1.53,2.47) |
| 5-variant GRS | 1 (Reference) | 1.12 (0.90,1.40 | 1.15 (0.92,1.43 | 1.39 (1.11,1.75) |
| **HR (95% CI) of total VTE ^‡^ for White participants**  273-variant GRS | 1 (Reference) | 1.24 (0.94,1.63) | 1.66 (1.26,2.19) | 2.12 (1.61,2.79) |
| 5-variant GRS | 1 (Reference) | 1.03 (0.78,1.37) | 1.14 (0.87,1.51) | 1.45  (1.10,1.92) |
| **HR (95% CI) of total VTE ^‡^ for Black participants**  273-variant GRS | 1 (Reference) | 1.01 (0.60,1.69) | 1.30 (0.79,2.13) | 1.24 (0.73,2.11) |
| 5-variant GRS | 1 (Reference) | 1.35 (0.93,1.96) | 1.18 (0.81,1.72) | 1.21  (0.81,1.81) |

*At baseline in 1987-89

**^‡^**Model 1: Adjusted for age, sex, and principal components of ancestry.
