# Supplementary figures and images for: Prediction of venous thromboembolism incidence in the general adult population using two published genetic risk scores
Source: PLoS One. 2023 Jan 30;18(1):e0280657. doi: 10.1371/journal.pone.0280657 (PMC9886242; doi:10.1371/journal.pone.0280657)

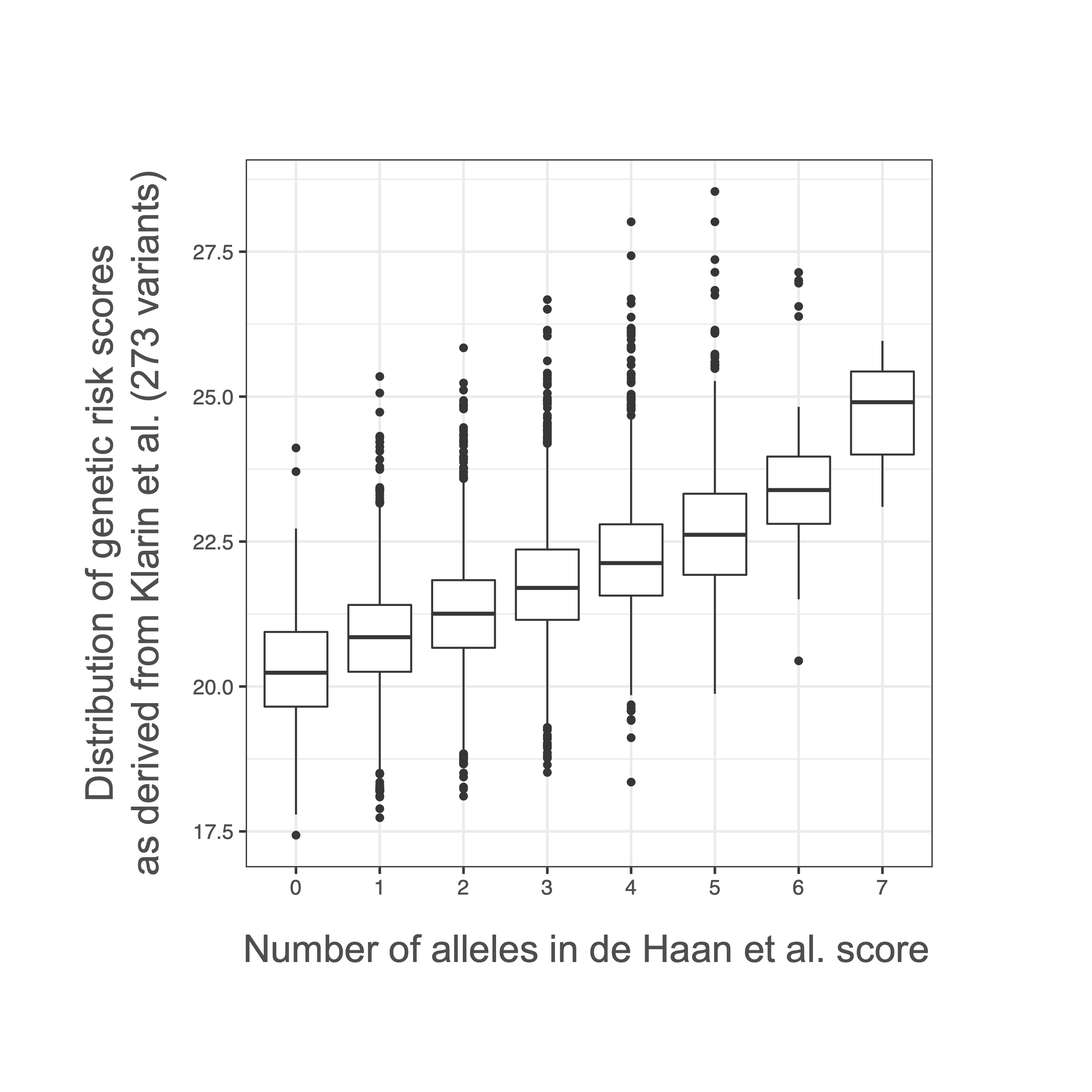

Supplement: S1 Fig — (TIFF) [file pone.0280657.s004.tiff]

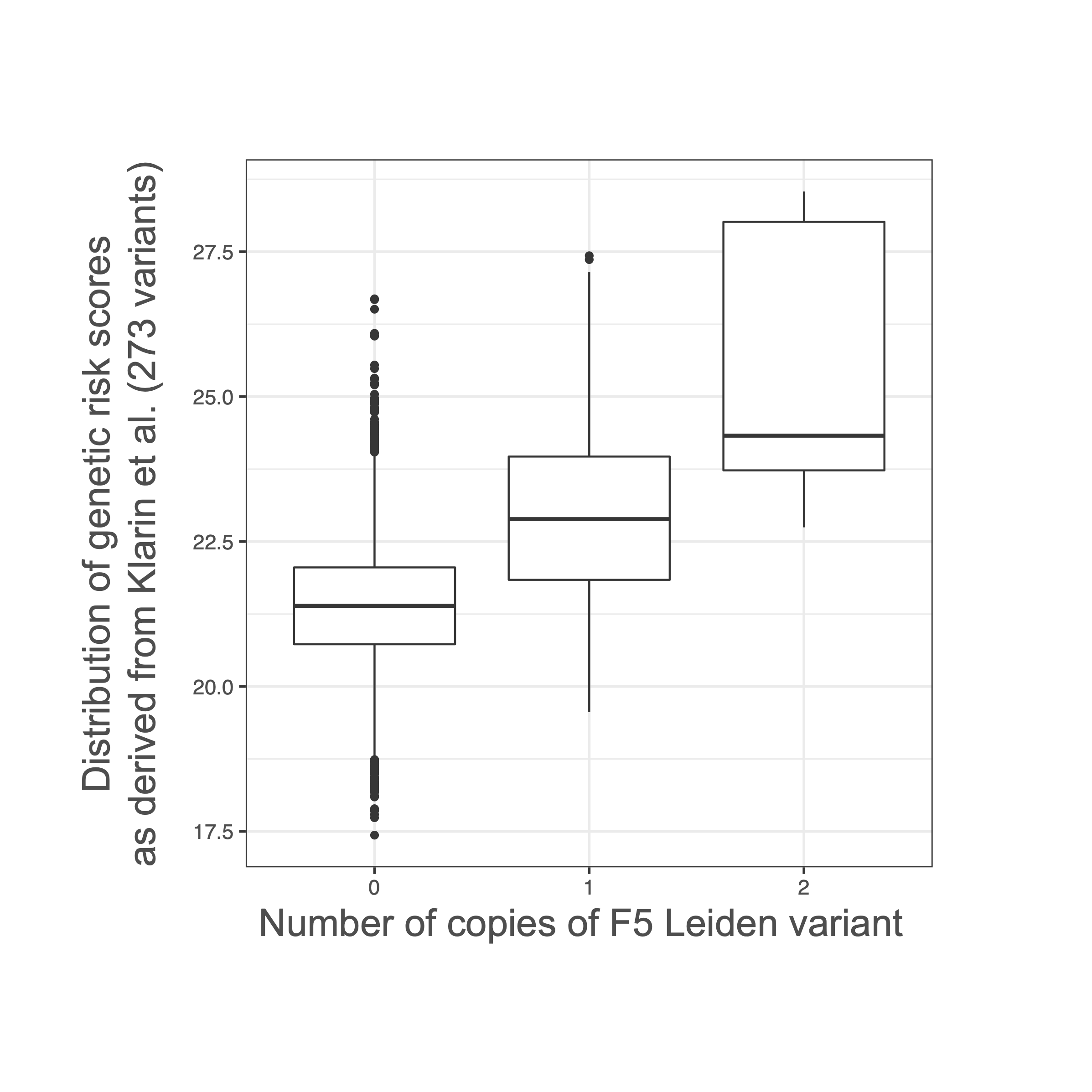

Supplement: S2 Fig — (TIFF) [file pone.0280657.s005.tiff]
