# Supplementary material for: Prediction of venous thromboembolism incidence in the general adult population using two published genetic risk scores
Source: PLoS One. 2023 Jan 30;18(1):e0280657. doi: 10.1371/journal.pone.0280657 (PMC9886242; doi:10.1371/journal.pone.0280657)

**Supplemental Figure S3**. Cumulative incidence of venous thromboembolism (VTE) by quartiles of (a) the 273-variant genetic risk score (GRS) or (b) the 5-variant GRS, ARIC, 1987-2019

(a) 273-variant


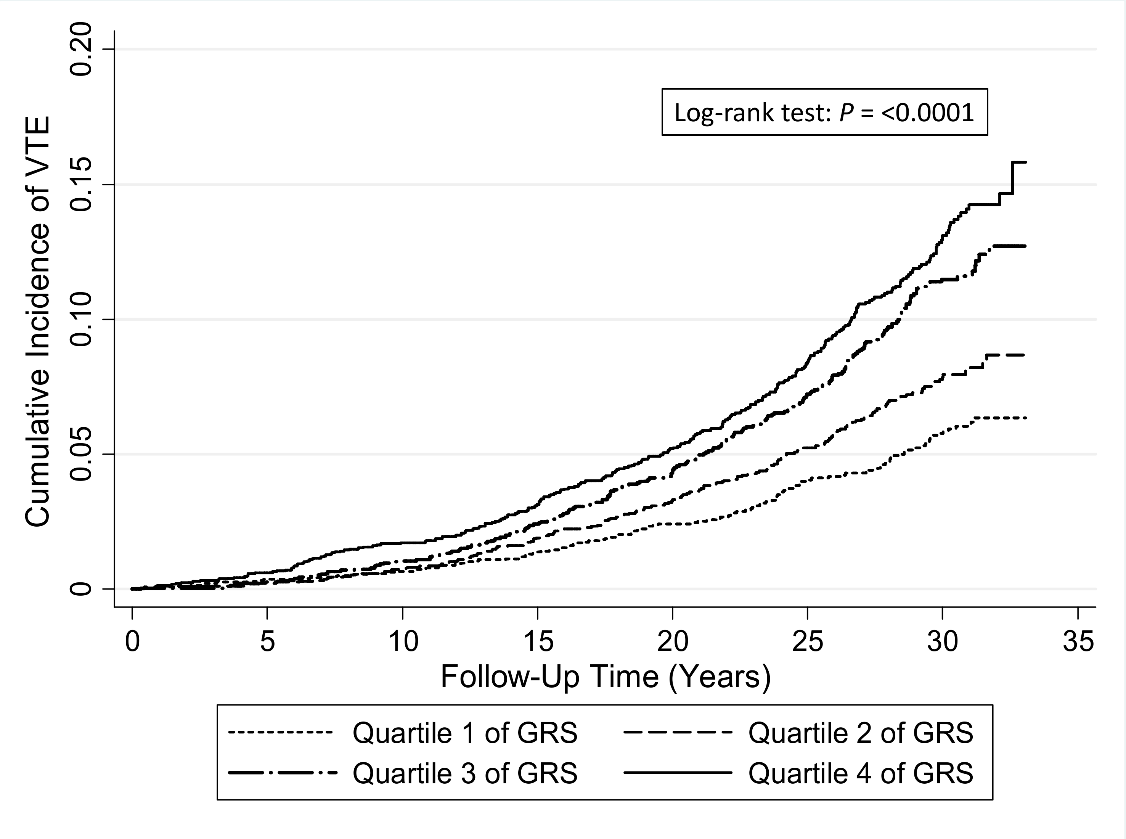


(b) 5-variant


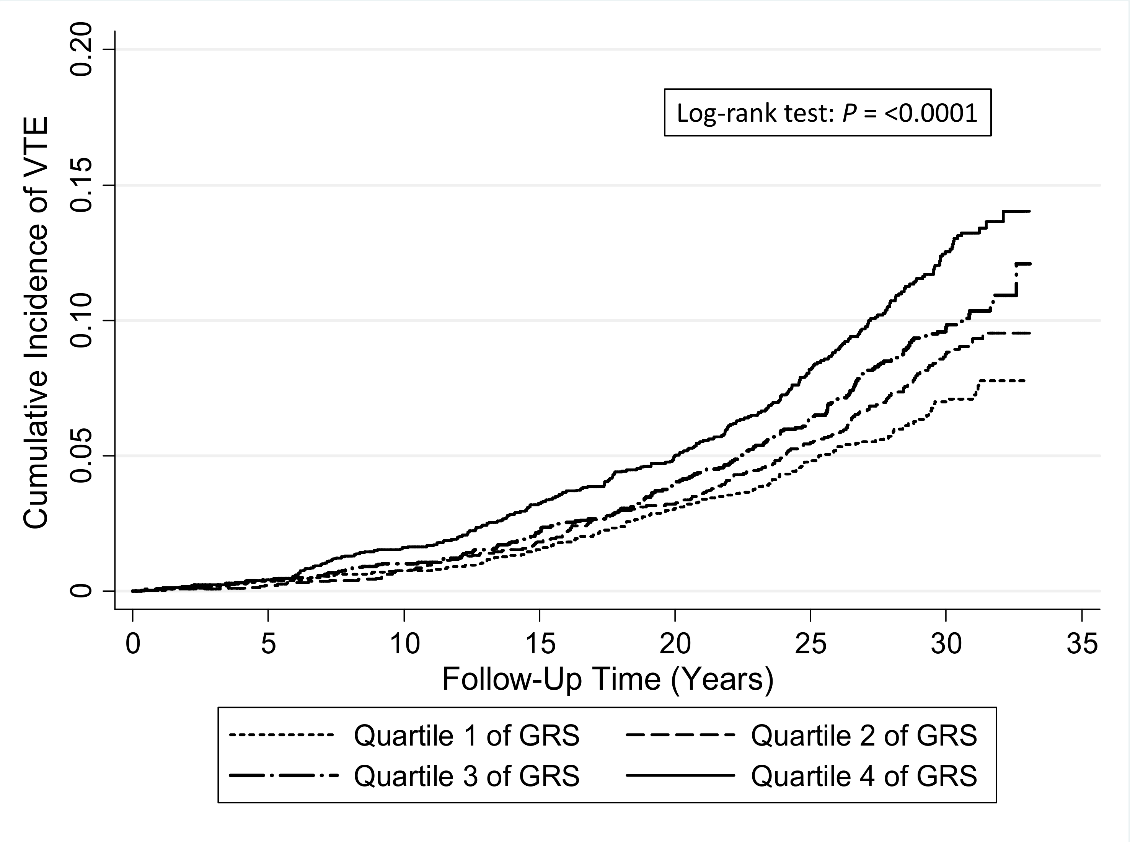

Supplement: S3 Fig — (DOCX) [file pone.0280657.s006.docx]
